# Bribery and the Role of Public Service Motivation and Social Value Orientation: A Multi-Site Experimental Study in Belgium, Germany and the Netherlands

**DOI:** 10.3389/fpsyg.2021.655964

**Published:** 2021-06-07

**Authors:** Lode De Waele, Kristina S. Weißmüller, Arjen van Witteloostuijn

**Affiliations:** ^1^Erasmus Hogeschool Brussel, Brussel, Belgium; ^2^Faculty of Social Sciences, University of Antwerp, Antwerp, Belgium; ^3^KPM Center for Public Management, University of Bern, Bern, Switzerland; ^4^Faculty of Business, Economics and Social Sciences, University of Hamburg, Hamburg, Germany; ^5^School of Business and Economics, VU University Amsterdam, Amsterdam, Netherlands; ^6^Faculty of Business and Economics, Antwerp University, Antwerp Management School, Antwerp, Belgium

**Keywords:** bribery, corruption, social value orientation (SVO), public service motivation (PSM), multi-site design

## Abstract

Bribery is a complex phenomenon rooted in both individual motives and the greater institutional context. Experimental research into causal mechanisms that drive bribing behavior is still scarce. To date, there is no empirical evidence on how the society-regarding motivational survey measure of Public Service Motivation (PSM) and the other-oriented motivational measure of Social Value Orientation (SVO) can help explain why some people are more susceptible to engage in the act of bribing than others. Based on a multi-site triple-replication, and a vignette-based research design, quasi-experimental evidence from Belgium, Germany, and the Netherlands shows that both measures interact and that—paradoxically—people with higher SVO are more likely to be willing to engage in bribery.

## Introduction

Bribery is still a wicked problem that does not seem to go away so easily, or at all, in many countries across the world. Bribery comes with very high social costs, undermining the sense of fairness. Bribery has macro-, meso-, and micro-level roots. First, a macro lens is required to explain differences across countries. Clearly, a society’s institutional context matters (Montinola and Jackman, 2002; Adelopo and Rufai, 2020). Second, in case of organization-level bribery, a meso lens is needed as organizational factors are argued to play an important role as well (den Nieuwenboer and Kaptein, 2008; Shaheer et al., 2019). Third, a micro perspective is required to understand individual variation within countries and across organizational contexts, as not all people equally engage in bribery within the same institutional and organizational environment (Navot et al., 2016). In the literature, the macro, meso, and micro perspectives live, by and large, separate lives in different disciplinary silos. For example, in Sociology, the importance of the institutional context for explaining bribery is emphasized; in Psychology, individual characteristics and motives are examined as the key drivers of bribery; and in Organization Studies, the main focus is on the meso-level roots of bribery.

The primary aim of this study is to contribute to the micro lens in a three-country study examining whether an individual’s Public Service Motivation (PSM) and Social Value Orientation (SVO) can help explain why some people are more likely than others to engage in bribery (Kwon, 2014). By including PSM and SVO, we link a well-established construct from Public Administration (PSM) with modern motivational theory in Social Psychology (SVO). As we will argue in greater detail below, PSM is an explicit survey measure of a critical motivational attitude regarding contributing to society at large, whilst SVO is an individual’s motivational other-regarding trait. We develop hypotheses as to how both characteristics in isolation and in tandem can be expected to affect the likelihood that an individual will engage in an act of bribery.

Specifically, the current study reports findings of a between-subject randomized vignette-based quasi-experiment regarding bribery within student samples from universities in Belgium (*n* = 220), Germany (*n* = 211), and the Netherlands (*n* = 193). The three treatments involve vignettes that differ in the seriousness of the bribery act in an educational setting in order to include sufficient contextual variation. The responses to these vignettes measure bribery willingness. We add a complementary questionnaire to measure key constructs that capture PSM and SVO as important potential micro determinants of bribery. The first, Public Service Motivation (PSM), is a concept that is central in Public Administration research. The second, Social Value Orientation (SVO), is a well-known notion in Social Psychology. In doing so, we develop a multidisciplinary theory combining the society-oriented construct of PSM with the individual-focused concept of SVO. By combining randomized vignettes with this pair of survey-based measures, we have a quasi-experimental design, with a treatment (bribery seriousness) and two individual characteristics (PSM and SVO), with willingness (to bribe) as the outcome.

In all, this article presents findings from three studies, replicating a novel quasi-experiment in three countries, examining the impact of PSM as a society-regarding motivational attitude and SVO as an other-oriented motivational value on bribery willingness. One aspect of the research design is worth emphasizing in advance, as this links to the macro perspective. Our study compares three countries that vary little in the macro incidence of bribery: Belgium, Germany, and the Netherlands. By design, we opted for the method of agreement as we aim to explore the replicability of our findings in similar contexts (Hilton et al., 1995; Walker et al., 2019). This results in a multi-site research design with three country contexts sufficiently alike to conduct replications without dominant contextual noise that would threaten comparability (Walker et al., 2019). This macro perspective is reflected upon in the discussion section, offering a limited discussion of a few cross-country differences to explore *post hoc* the macro lens, suggesting interesting avenues for future research. As a side-benefit, by running the quasi-experiment in three countries, this research responds to the appeal to conduct replication studies (see, e.g., van Witteloostuijn, 2016; Walker et al., 2019). By doing so, we can reflect on the generalizability of the findings, including a discussion of the boundary conditions of our theory.

## Theory

### Bribery

Bribery is a multi-facetted and many-faced phenomenon. We start from the Cambridge Dictionary’s common sense definition as “an attempt to make someone do something for you by giving the person money, presents, or something else that they want.” Heidenheimer (2009) distinguishes three *shades* of bribery: Black bribery, gray bribery, and white bribery. Black bribery is a particular action that by majority consensus of public opinion should be condemned and punished on grounds of principle. Gray bribery implies that limited elements in society, usually elites, may want to see the actions punished and that the majority may well be ambiguous. White bribery is tolerated by the majority of both elite and mass opinion, and attempts to punish this form of bribery are not likely to find public support (Heidenheimer, 2009).

According to Ramdani and van Witteloostuijn (2014), bribery is defined as “the corrupt payment, receipt, or solicitation of a private favor for actions or decisions from influential or powerful agents or authorities which could be public officials, corporations or people inside corporations to generate private benefits of the briber.” Cultural and institutional differences across countries and regions play an important role in determining the incidence of bribery (Martin et al., 2007; Li et al., 2008). For instance, bribery is usually found to be high in countries with limited political competition and low GDP per capita (Montinola and Jackman, 2002; Wilhelm, 2002). Furthermore, the organizational context plays an important role in explaining bribery, as bribery has been found to be especially likely in highly competitive contexts in which managers fail to correct corrupt behavior and in situations in which employees feel pressured to ward off identity threats to the organization (den Nieuwenboer and Kaptein, 2008).

However, the likelihood of bribery cannot be explained exclusively by referring to the macro or meso environment. Prior research by, for instance, Martin et al. (2007), Jávor and Jancsics (2016), and Ramdani and van Witteloostuijn (2012a,b) demonstrates the critical importance of individual micro attributes. Individual characteristics such as age, gender and education, but also personal risk preferences are argued to have an effect on the likelihood that an individual person will offer and/or accept bribes (Alatas et al., 2009; Nichols and Robertson, 2017). In this context, the potential effect of a key construct in Public Administration research—Public Service Motivation (PSM)—has not been examined in great detail, to date. Yet, PSM is argued to be a critical determinant guiding behavior aimed to benefit communities or societies at large (Esteve et al., 2015; Kim and Kim, 2016; van Witteloostuijn et al., 2017). As a starting point, therefore, we first theorize how PSM might impact the likelihood that an individual will engage in the act of bribing.

### Public Service Motivation

Public Service Motivation (PSM) is one of the most prominent concepts in both Public Administration and Public Management research. As one of the pioneers of the PSM concept, Perry (1996) describes PSM as “an individual’s predisposition to respond to motives grounded primarily or uniquely in public institutions.” PSM originally consists of six motives: Civic duty, social justice, commitment to the public interest, self-sacrifice, compassion, and attraction to public interest (Perry, 1996). The concept of PSM has evolved greatly over time, with much scholarship examining PSM’s antecedents, definitions, consequences, and measures (Bozeman and Su, 2015). For instance, Coursey and Pandey (2007), Vandenabeele (2008), Kim (2009), and Esteve et al. (2016) consolidated PSM, shaping its current and most widely used four-dimensional form, comprising attraction to policy-making (APM), commitment to public interest (CPI), compassion (COM), and self-sacrifice (SS).

The potential link between PSM and bribery has been referred to in conceptual, normative, and theoretical terms. However, to date, a detailed empirical study is yet to be conducted (Kim and Kim, 2016). From a theoretical perspective, the argument is very straightforward and highly intuitive. After all, by the very definition of the construct, the expectation is that high-PSM people prioritize serving the public interest, even to the extent of sacrificing their own self-interest. This motive strongly goes against any form of bribery that involves serving self-interest at the expense of the public. Indeed, the literature has argued that people scoring high on PSM are, on average, more sensitive to unfair competition and do strongly oppose unethical behavior, of which bribery is a clear example (Kwon, 2014; Kim and Kim, 2016; Wright et al., 2016; Wang and Seifert, 2020). This gives our intuitive baseline hypothesis.

*HYPOTHESIS 1 (H1): The relationship between public service motivation and the likelihood to engage in bribery is negative, implying that individuals’ likelihood of engaging in bribery decreases with higher levels of public service motivation.*

### Social Value Orientation

Traditional normative theories of behavior assume that (i) people are rational decision-makers and that (ii) they are mainly motivated by self-interest (von Neumann and Morgenstern, 1944; Luce and Raiffa, 1957). Yet, subsequent theoretical advancements indicate that individuals systematically differ in the manner in which they interact with independent others (Kuhlman and Marshello, 1975; van Lange and Kuhlman, 1994). Fundamental psychological work such as that of Bogaert et al. (2008) and Balliet et al. (2009) shows that this systematic divergence from pure self-serving behavior is related to individuals’ Social Value Orientation (SVO). This concept, according to Messick and McClintock (1968), refers to a stable preference for certain outcomes for oneself against others’ outcomes, thus capturing the extent to which an individual is intrinsically mainly concerned with personal versus group well-being. In the SVO literature, many scholars use an ideal-typical binary categorization, pro-self-vis-à-vis pro-social (but see Murphy and Ackermann, 2014). On the one hand, pro-self people knowingly or subconsciously work toward the realization of their personal goals with little or no regard to other peoples’ goals, whereas, on the other hand, their pro-social counterparts also consider the goals of others, attaching more importance to the well-being of a community or society as a whole.

Social value orientation is strongly related with pro-social and ethical behavior. According to Batson and Powell (2003), pro-social behavior covers a wide range of actions intended to benefit one or more people other than oneself, such as caring, helping, comforting, sharing, and cooperating. These behaviors can benefit family, coworkers, customers, teams, stakeholders or an organization or community as a whole (Bolino and Grant, 2016). Pro-social behavior is positively linked with individual organizational outcomes such as job performance, organizational commitment, and career success. Based on processes of reciprocity, such behavior creates strong and weak ties, leading to the construction of social capital (Bolino et al., 2002; Shah et al., 2018). Furthermore, pro-social individuals are perceived as being less threatening to others, which makes them valued allies instead of competitors (Casciaro and Lobo, 2008; Bendell, 2017). De Cremer and van Lange (2001), in line with earlier research by Sattler and Kerr (1991), argue that high-SVO people reveal a greater concern for others and for the group, and that they judge more in terms of non-egoistic values such as fairness, honesty, and equality.

The above logic has a clear implication relevant for understanding the relation between SVO and the likelihood to engage in self-focused bribery, very similar to that of PSM. Basically, pro-self individuals will be more likely to engage in bribery to serve their self-interest than their pro-social counterparts, given that the very definition of bribery implies an act of self-interest. Hence, SVO is expected, as is overall PSM, to be negatively related to the likelihood of engaging in bribery. Consequently, we have our second baseline hypothesis.

*HYPOTHESIS 2 (H2): The relationship between social value orientation and the likelihood to engage in bribery is negative, implying that individuals’ likelihood of engaging in bribery decreases with higher levels of social value orientation.*

### PSM and SVO in Tandem

Taking Public Administration’s PSM and Social Psychology’s SVO together, we can identify two further channels of influence on an individual’s bribery likelihood. The first is that SVO, as an other-regarding motivational value, may moderate the impact of PSM, as a society-regarding motivational attitude. On the one hand, in Public Administration, an ongoing debate revolves around the question as to whether PSM is an individual attitude or trait, or a combination of the two, with clear evidence that the attitudinal dimension is prominent (see, e.g., van Witteloostuijn et al., 2017). On the other hand, in line with modern motivational theory in Psychology, SVO is seen as an individual trait (see, e.g., Grant, 2008). Hence, we could argue that the attitudinal effect of high PSM is stronger if supported by a trait-driven “multiplier” of a pro-other SVO. That is, if an individual indicates s/he is motivated to serve the public interest, which includes a general motivation to self-sacrifice for the sake of others’ benefit, then this motivational attitude is further boosted if this individual is characterized by the other-regarding trait of SVO as well.

*HYPOTHESIS 3 (H3): Social value orientation positively moderates the relationship between public service motivation and the likelihood to engage in bribery.*

The second additional channel of influence involves mediation. Implicitly, our hypotheses above suggest a specific relationship between a society-regarding attitude (PSM) and an other-regarding trait (SVO) motive, the latter mediating the effect of the former. Human motives are defined as someone’s capacity to experience a specific type of stimulus, incentive or activity as pleasurable, hence directing behavior (Schultheiss and Brunstein, 2010). Such motives, which can be explicit or implicit, involve stable differences in desires from which people derive pleasure (McClelland et al., 1989). Here, combining this with PSM, we see a parallel with motivational theory’s argument that some (explicit) motives “push” individuals toward actions that they enjoy (“want-to” behavior), whereas other (implicit) motives “pull” them toward actions that they feel obliged to (“have-to” behaviors) (Kehr, 2004; Hermans et al., 2017). Similarly, we would argue that a contextual pull to serve the public interest due to high PSM is triggered further through a “push” running through an individual’s other-regarding value due to high SVO.

*HYPOTHESIS 4 (H4): Social value orientation partially mediates the relationship between public service motivation and the likelihood to engage in bribery.*

**FIGURE 1:**
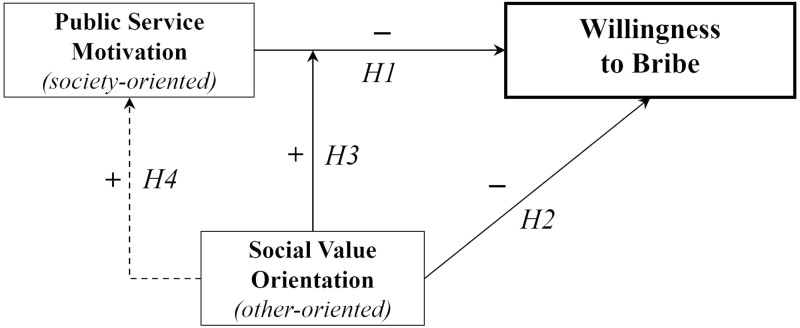
Conceptual framework.

The full model is summarized in Figure 1.

Note that we have no *a priori* predictions regarding sub-dimensions of PSM (see above) or sub-aspects of bribery willingness (see below), given lack of any extant work on this. However, inductively, we will conduct exploratory analyses to identify any meaningful finer-grained relationship.

## Methods

### Multi-National and Multi-Site Vignettes Experiment

An original multi-site vignettes quasi-experiment was conducted as an online survey with three independent country samples from April to August 2017. This experiment was programmed and hosted with Qualtrics’ software, and distributed *via* e-mail invitation among students of four university faculties in Belgium, Germany, and the Netherlands, across a variety of economic and social sciences, ranging from public and for-profit management, to political sciences, socio-economic studies, and business engineering. Participation was voluntary. In each country, students were incentivized with the possibility of winning one of four significant (1 x €250, 1 x €150, and 2 x €50) gift certificates for a well-known online retailer. The English scale items were translated into Dutch (for both Belgium and the Netherlands) and German, and slightly adapted to the local context to accommodate the specific national conditions of higher education. The dataset was strictly pre-stratified for missing and repetitive responses, and comprises only complete non-skewed (centralized) responses. In the prospect of small effect sizes (Cohen’s *d* ≤ |0.3|; powe*r* = 0.8; α = 0.05), conservative estimates prior to data collection indicate that the necessary absolute sample size requires *n* = 176 respondents per study (Ellis, 2010). This has been achieved.

Vignettes are stimuli in the form of narrative and realistic scenarios in which participants are invited to imagine that they have to act in the vignette’s context, and respond accordingly to a series of survey items (Hughes and Huby, 2004). Vignettes are proven to be very useful treatment instruments in experiments with the power to systematically manipulate and trigger context-dependent behavior at high degrees of both internal and external validity (Aguinis and Bradley, 2014). The quasi-experiment (see Supplementary Appendix A.1 for detailed procedures and vignettes) is composed of four parts: (1) A short introduction; (2) a socio-demographic questionnaire with control variables to warrant sample balancing; (3) standardized measures of PSM and SVO (our key independent variables); and (4) a randomized vignettes treatment.

### Independent and Control Variables

Central to our design are PSM and SVO, being a society-regarding attitudinal and an other-regarding value motivation measure, respectively. PSM can be easily measured *via* a well-validated self-report questionnaire, whereas SVO is frequently captured through a well-established forced-choice revealed preference survey measure. First, following Esteve et al. (2015, 2016), PSM was measured with Kim et al.’s (2013) twelve-item Likert-type scale in its four-dimensional conceptualization (see Supplementary Appendix A.2 for all items and psychometrics, per sample). The scale items were translated with a double-blind back-translation procedure to maximize reliability. Items must be scored from 1 = “absolutely disagree” to 7 = “absolutely agree.” The Cronbach’s α is 0.82 for the Belgian (AIC = 0.462), 0.87 for the German (AIC = 0.693), and 0.84 for the Dutch sample (AIC = 0.564), and 0.84 for the pooled data. We use mean sum scores for PSM overall (pooled data: Cronbach’s α = 0.84; AIC = 0.571; AVE = 0.323; Chi^2^ (54) = 54,346; *p* < 0.000; GFI = 0.731; RMSEA = 0.141; CFI = 0.731).

Social value orientation was measured with Bogaert et al.’s (2012) validated procedure, which is based on a well-established forced-choice technique to capture revealed preferences (Supplementary Appendix A.3). Respondents have to make decisions over a total of nine scenarios based on the classic Dictator Game setup, in which they decide about what proportion of a hypothetical reward of, on average, €500 they are willing to share with another anonymous person. Each scenario offered three systematically varied choice options that are each characteristic for either a competitive choice (e.g., €480 for self and €80 for other), individualistic choice (e.g., €540 for self and €280 for other), or pro-social choice (e.g., €480 for self and €480 for other) (Kuhlman and Marshello, 1975; Bogaert et al., 2012). Counting the number of pro-social choices, this measure results in a compound ranking score ranging from 0 to 9 for each participant, in which higher scores indicate higher SVO. This caters to the critique of categorized measures of SVO (Murphy and Ackermann, 2014).

Intuitively, PSM and SVO are related constructs, both rooted in the motivation to care for others. Theoretically, above we argue and assume that both constructs are different, PSM being attitude-based and society-regarding, and SVO being trait-based and other-regarding. Indeed, the nature of both measures is very different, with PSM being measured with an attitudinal questionnaire scale, and SVO with revealed preferences in the form of a series of forced-choice scenarios. So, the question is to what extent both measures are indeed different empirically. In our three sub-samples and the pooled sample, they clearly are, with *r* = −0.40 (*p* = 0.000) in the Belgian, *r* = −0.435 (*p* = 0.000) in the Dutch, and *r* = −0.394 (*p* = 0.000) in the German sub-sample, aggregating into *r* = −0.412 (*p* = 0.000) in the pooled sample. Empirically, these negative Pearson correlation values confirm our expectation that our two measures tap into two different constructs, implying that we can run meaningful statistical analyses.

As a key control variable, participants’ risk attitude was assessed with Madden et al.’s (2009) Probability Discounting Questionnaire, a measure that estimates revealed behavior under risk based on responses to a systematic and randomized set of economic trade-off tasks, which result in a metric discounting parameter *ln*(h) for each individual (see Weißmüller (2021) for more detail and the aggregation algorithm). Compared with stated preferences, revealed behavior is a more reliable indicator for people’s actual behavior outside the study context. Since higher discounting parameters indicate stronger probability discounting, individuals with *ln*(h) > 0 are characterized as risk averse. The other control variables are the usual socio-demographic suspects (see Table 1 for details): respondent’s age, gender, and religiosity. Finally, we control for country-of-origin, as our main analysis is performed with the data pooled over our three country samples (see below).

**TABLE 1:** Descriptive statistics.

	Study 1	Study 2	Study 3
Sampling site	Belgium	Germany	The Netherlands
*N*	220	211	193
**Experimental treatment**			
White bribery (%) ^*a*^	34.7	33.8	34.2
Gray bribery (%) ^*a*^	34.8	33.8	31.4
Black bribery (%) ^*a*^	34.9	34.2	30.8
**Motivation**			
**Society-oriented:**			
Public Service Motivation (PSM); *M* ± *SD*	5.53 ± 0.85	5.26 ± 0.98	5.38 ± 0.92
Attraction to Policy Making	5.94 ± 1.14	5.47 ± 1.32	5.86 ± 1.06
Commitment to Public Interest	5.72 ± 1.03	5.52 ± 1.13	5.39 ± 1.15
Compassion	5.60 ± 1.15	5.61 ± 1.14	5.55 ± 1.05
Self-Sacrifice	5.18 ± 1.17	4.73 ± 1.19	5.01 ± 1.36
**Other-oriented:**			
Social Value Orientation (SVO); *M* ± *SD*	6.51 ± 3.57	4.83 ± 2.93	6.11 ± 3.76
**Socio-demographic characteristics**			
Gender, male (*n*) ^*a*^	48.2% (104)	45.2% (95)	48.2% (93)
Age in years ^*a*^	22.47 ± 3.65	25.84 ± 4.82	21.13 ± 2.82
Risk aversion (revealed) ^*b*^	1.57 ± 0.65	0.65 ± 0.62	0.96 ± 0.61
**Nationality**			
Belgian	89.1% (196)	.	.
German	.	88.6% (187)	3.1% (6)
Dutch	8.6% (19)	.	93.3% (180)
Other	2.3% (5)	11.4% (24)	3.6% (7)
Religiosity, yes (*n*)	50.5% (111)	59.2% (125)	32.6% (63)

*Items are either reported with geometric means and standard deviations (*M* ± *SD*) or proportions (%) and frequencies (*n*); ^*a*^treatment distribution checked for balance with two-tailed *t*-tests within and between studies; all non-significant; ^*b*^logarithmic probability discounting parameter, centralized.*

### Experimental Treatment Vignettes

Respondents were randomly assigned to two out of three bribery vignettes, which were designed with due diligence following Hughes and Huby (2004). Treatment randomization is an essential requirement for (quasi-) experimental research seeking to infer causal relations (Jilke and Van Ryzin, 2017). The balance between treatment groups was strictly controlled for, with success (see Table 1). The vignettes were designed to represent Ramdani and van Witteloostuijn’s (2014) three shades of bribery, ranging from white *via* gray to black forms of bribery. They comprise realistic scenarios in which respondents were in the active role of a student proposing a bribery offer to a professor in exchange of reconsideration of an important exam score. The external validity of this approach was corroborated with an expert panel, as suggested by Gould (1996). Furthermore, adequate pre-tests with students and focus groups were conducted in the process of pretesting the survey items with a relevant pilot sample (Wilson and While, 1998).

In the white bribery vignette, the student does not offer anything material to the teacher, but only engages in an emotional plea. This vignette may even be argued to be so white that this does not involve bribery at all. But still, the student does ask the teacher to engage in an act—i.e., adjusting a grade upward—that is taken to be unethical in many countries, and certainly so in Belgium, Germany, and the Netherlands. In the gray bribery vignette, the bribery act does come with a material favor, offering to take care of a car repair for free. In the Belgian, Dutch, and German higher university context, this clearly comes down to a bribery act, albeit one which does not involve an actual exchange of money. In the black bribery vignette, the latter is offered, which is a considered a clear and unambiguous example of bribery in our set of three countries. And what is crucial in the context of our treatment effect, the three vignettes can clearly be ranked in order of the seriousness of the involved offer, from weak (white) *via* medium (gray) to strong (black).

### Dependent Variables

We developed a novel measure for respondents’ willingness to engage in bribery, which we refer to as *Willingness to Bribe* (WtB). WtB and its underlying components (see below) serve as our main dependent variables. After each vignette treatment, respondents were asked to indicate how they would react in this specific scenario by answering four Likert-type items ranging from 1 = “absolutely disagree” to 5 = “absolutely agree.” These four items were designed to load onto respondents’ attitudes regarding bribing the professor in the respective scenarios—by asking how likely they were to bribe in this context (*likelihood*), how justified offering the bribe was (*justification*), how comfortable they would feel in doing so (*affect*), and whether offering a bribe would be a mistake (*mistake*), which was a reverse item for control. These four factor items are, first, analyzed as separate components, but are in subsequent steps of the analysis geometrically sum-scored to form WtB.

The validity of this aggregation procedure was tested with exploratory factor analysis (varimax rotated with Kaiser normalization for item correlation), which confirmed very high internal validity and robustness against country effects when repeated separately for each of the three study samples (see Supplementary Appendix A.4). The resulting dependent variable is normally distributed across all treatment conditions (shades of bribery) [tested with Shapiro-Wilk; vignette 1: W(409) = 0.991, *p* = 0.015; vignette 2: W(417) = 0.954, *p* = 0.000; vignette 3: W(415) = 0.892, *p* = 0.000], and thus allows for linear regression analysis. As a control variable, respondents were asked to rate how realistic they found the scenario on a four-point Likert-type single item, ranging from 1 = “very unrealistic” to 4 = “very realistic.” We control for balanced perceived vignette scenario realism—as a proxy for verisimilitude of the treatment condition—to guarantee high ecological validity of the estimation model.

### Model Estimation

**FIGURE 2:**
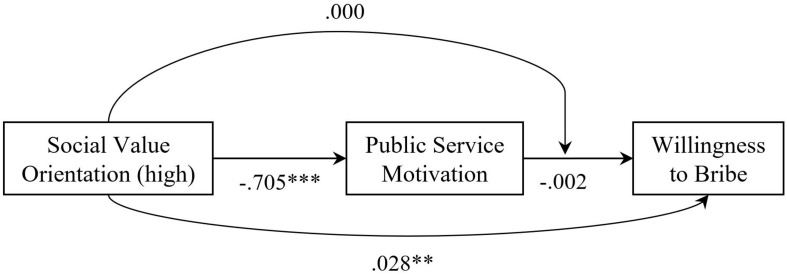
Results of SEM analyses. Results of structural equation modeling, describing direct and indirect paths; based on pooled data by *N* = 622 respondents (*n* = 1,242 observations); ^∗∗^*p* < 0.01, ^∗∗∗^*p* < 0.001.

Because study participants always responded to two vignettes, linear regression analysis was conducted with heteroscedasticity-robust standard errors clustered at the subject level to account for latent vignette clustering effects. The dependent variable is aggregated WtB or the four components of WtB, respectively. As we explain below, we pooled the data across our three country samples. Subsequently, in our main analysis, we run three models, as reported in Table 2: Models I include control variables only, Models II add PSM and SVO to test our main effect hypotheses (H1 and H2), and Models III explore the PSM^∗^SVO moderation effect (H3). White bribery and the Belgian sample serve as arbitrary reference categories. In a second exploratory modeling step, we investigate the effect of each of the four classic dimensions of PSM by including APM, CPI, COM, and SS separately in the split and pooled regression models presented in the online Supplementary Appendix Table A.5.1 in Supplementary Appendix A.5, to safe space. Finally, we conduct a moderated mediation test, reported in Figure 2, to find out to what extent, if at all, PSM operates as a partial mediator in the PSM—WtB relationship (H4), conditional on PSM^∗^SVO moderation.

**TABLE 2:** Regression estimates on Willingness to Bribe (pooled data).

	Likelihood			Justification			Affect			Mistake			Willingness to Bribe		
					
	I	II	III	I	II	III	I	II	III	I	II	III	I	II	III
Public Service		−0.042	−0.014		−0.045	−0.057		−0.057	−0.052		0.103**	0.077		−0.013	−0.021
Motivation		(0.035)	(0.042)		(0.037)	(0.050)		(0.039)	(0.044)		(0.044)	(0.063)		(0.019)	(0.026)
Social Value		0.196**	0.479		0.177**	0.053		0.167**	0.219		−0.172**	−0.441		0.124***	0.056
Orientation		(0.067)	(0.366)		(0.066)	(0.412)		(0.062)	(0.416)		(0.076)	(0.482)		(0.040)	(0.219)
Public Service			−0.053			0.023			−0.010			0.050			0.015
Motivation * Social Value Orientation			(0.068)			(0.075)			(0.077)			(0.088)			(0.041)
Age	0.000	0.005	0.005	0.014^†^	0.019*	0.019*	−0.000	0.004	0.004	−0.023**	−0.029**	−0.030**	−0.002	−0.000	0.000
	(0.007)	(0.007)	(0.007)	(0.008)	(0.008)	(0.008)	(0.007)	(0.007)	(0.007)	(0.010)	(0.010)	(0.010)	(0.004)	(0.004)	(0.004)
Female	−0.146**	−0.112^†^	−0.111^†^	−0.181**	−0.149*	−0.150*	−0.271***	−0.237***	−0.236***	0.144*	0.098	0.097	−0.143***	−0.124***	−0.124***
	(0.064)	(0.064)	(0.064)	(0.061)	(0.061)	(0.061)	(0.057)	(0.056)	(0.057)	(0.073)	(0.072)	(0.073)	(0.036)	(0.036)	(0.036)
White bribery	0.415***	0.430***	0.433***	0.369***	0.383***	0.381***	0.146*	0.161*	0.162*	−0.492***	−0.512***	−0.516***	0.143**	0.151**	0.150***
	(0.078)	(0.077)	(0.077)	(0.074)	(0.074)	(0.074)	(0.072)	(0.070)	(0.070)	(0.092)	(0.091)	(0.090)	(0.045)	(0.044)	(0.044)
**Gray bribery**							*- reference category -*								
Black bribery	−0.132^†^	−0.133^†^	−0.131^†^	−0.029	−0.029	−0.030	−0.025	−0.027	−0.026	−0.076	−0.072	−0.074	−0.103*	−0.103*	−0.103*
	(0.080)	(0.079)	(0.079)	(0.072)	(0.072)	(0.072)	(0.063)	(0.063)	(0.063)	(0.085)	(0.084)	(0.084)	(0.042)	(0.042)	(0.042)
Germany	−0.083	−0.106	−0.104	0.167*	0.143^†^	0.143^†^	−0.067	−0.095	−0.095	−0.155	−0.110	−0.111	−0.026	−0.036	−0.037
	(0.081)	(0.081)	(0.081)	(0.085)	(0.083)	(0.083)	(0.073)	(0.071)	(0.072)	(0.102)	(0.101)	(0.101)	(0.047)	(0.046)	(0.046)
**Belgium**							*- reference category -*								
The Netherlands	0.034	0.028	0.037	−0.149*	−0.155*	−0.155*	−0.020	−0.028	−0.028	0.074	0.089	0.090	−0.025	−0.026	−0.026
	(0.076)	(0.075)	(0.075)	(0.066)	(0.066)	(0.066)	(0.068)	(0.068)	(0.068)	(0.079)	(0.078)	(0.078)	(0.044)	(0.043)	(0.043)
Intercept	1.819***	1.837***	1.675***	1.470***	1.521***	1.592***	1.830***	1.944***	1.914***	4.643***	4.319***	4.473***	2.155***	2.099***	2.144***

	(0.198)	(0.277)	(0.311)	(0.204)	(0.292)	(0.357)	(0.189)	(0.296)	(0.309)	(0.261)	(0.363)	(0.466)	(0.000)	(0.163)	(0.192)
*F*	10.97	10.73	9.54	11.85	11.86	10.75	5.46	7.02	6.25	9.46	9.83	8.81	1.31	9.24	8.35
*p*	0.000	0.000	0.000	0.000	0.000	0.000	0.000	0.000	0.000	0.000	0.000	0.000	0.035	0.000	0.000
*VIF ^*a*^*	1.31	1.29	9.79	1.31	1.29	9.78	1.31	1.29	9.79	1.31	1.29	9.79	0.58	1.29	9.78
*R*^2^	0.045	0.055	0.055	0.054	0.063	0.063	0.029	0.043	0.043	0.048	0.061	0.062	−0.002	0.059	0.059
*RMSE*	1.137	1.131	1.131	1.068	1.062	1.062	0.904	0.897	0.897	1.225	1.216	1.216	(0.004)	0.571	0.571

Linear regression modeling clustered by *N* = 622 respondents for conditional contribution (*n* = 1,242 observations), robust standard errors in parentheses; ^*a*^ Mean variance inflation factor (*VIF*), all *VIF* ≤ 1.58 in models I, all *VIF* ≤ 1.54 in models II; ^†^*p* < 0.10, *p < 0.05, **p < 0.01, and ***p < 0.001.

## Findings

### Descriptive Analysis

#### Belgium

The data of sample 1 was raised at the faculty for business and social sciences of a large public university in Belgium. The average time to completion was 13.6 min. The sample comprises *n* = 220 respondents, which are slightly dominated by female participants (51.8%), on average 22.5 (± 3.7) years old, predominantly risk-averse and non-religious (49.6%), and who study a variety of business and social sciences, especially Business Administration (46.8%). Respondents score relatively high on PSM (*M* = 5.53, *SD* = 0.85), especially on the dimensions of APM (*M* = 5.94, *SD* = 1.14) and CPI (*M* = 5.72, *SD* = 1.03), and they reveal high SVO (*M* = 6.51, *SD* = 3.57) (see Table 1 for more detail).

Across all treatments, respondents score below the scale’s medium on WtB (*M* = 1.87, *SD* = 0.91). The sample perceived the vignettes as above-average realistic (*M* = 2.10, *SD* = 0.77). Two-tailed *t*-testing shows that the vignettes create significant variance, with WtB strictly decreasing from the white (*M* = 2.65, *SD* = 0.87) to the black bribery scenario (*M* = 1.31, *SD* = 0.53), indicating a strong and robust treatment effect [*t*(287) = −15.781, *p* = 0.000, *d* = −1.863]. Pair-wise correlation analysis of the dependent variable by bribery vignette indicates high internal discriminant validity with very small inter-item covariance (0.067) and high construct validity of the dependent variable items (Cronbach’s α = 0.60), which confirms that the three different vignettes trigger the same underlying concept.

#### Germany

Respondents of study 2 are *n* = 211 students in economics and social sciences, especially Public Administration (*n* = 38) and Business Administration (*n* = 37), at a large public university in Germany. Respondents were, on average, 25.8 (± 4.8) years old, predominately female (54.8%), risk-averse, and non-religious (40.8%). Respondents report high PSM (*M* = 5.26, *SD* = 0.98), with relative high scores in COM (*M* = 5.61, *SD* = 1.14) and relative low scores in SS (*M* = 4.73, *SD* = 1.19). On the nine-point SVO scale, a mean value of 4.83 (± 2.93) is just slightly above the scale’s average. This sample’s response toward the realism item is above average (*M* = 2.23, *SD* = 0.85). The average time to experiment completion was 15.6 min. Across all treatment conditions, respondents score lower than average on WtB (*M* = 2.05, *SD* = 0.97). Two-tailed *t*-testing analysis indicates that the three vignettes resulted in sufficient variance in the bribery treatment. Respondents’ WtB decreases strictly and transitively the darker the shade of bribery from white (*M* = 2.65, *SD* = 0.95) to black bribery (*M* = 1.60, *SD* = 0.79), which indicates a strong and robust treatment effect [*t*(284) = −10.076, *p* = 0.000, *d* = −1.200]. Pairwise correlation analysis indicates high internal discriminant validity of the three treatment scenarios (AIC = 0.057) and satisfactory construct validity (Cronbach’s α = 0.45).

#### The Netherlands

The data of study 3 were collected at the faculties for business and social studies at two public universities in the Netherlands. The sample (*n* = 193) is slightly dominated by female (51.8%) and non-religious (67.7%) participants, with an average age of 21.1 (± 2.8) years, who are predominantly risk-averse. Respondents are mostly students of Business Administration (36.1%) or Socioeconomics (31.3%). Respondents score relatively high on the compound PSM scale (*M* = 5.38, *SD* = 0.92), especially high on APM (*M* = 5.86, *SD* = 1.06) and especially low but still clearly above average on SS (*M* = 5.01, *SD* = 1.36; seven-point scale). The sample reveals high average SVO (*M* = 6.11, *SD* = 3.76), and perceived the scenarios as realistic (across all vignettes: *M* = 2.05, *SD* = 0.78). The average time to completion of the experiment was 13.7 min. Over all treatments, respondents score medium high on WtB (*M* = 1.94, *SD* = 0.96). Participants’ response on WtB decrease strictly and transitively from the white (*M* = 2.69, *SD* = 0.90) to the black bribery treatment (*M* = 1.39, *SD* = 0.66), indicating a strong and robust treatment effect [*t*(255) = −13.088, *p* = 0.000, *d* = −1.639]. Pairwise correlation analysis shows that the three treatment conditions have high internal discriminant validity (inter-item covariance = 0.090) and high construct validity (Cronbach’s α = 0.61).

### Main Analysis

The three country-specific data sets were pooled to a total sample of *n* = 1,242 in order to test our hypotheses and account for country-specific effects. Furthermore, the larger *n* implies that the hypotheses can be tested with greater power then by analyzing individual country samples. All clustered regression models are well specified [*F*(df, 621) = 6.28—12.94, *p* < 0.000], and multicollinearity was not an issue (mean *VIF* = 1.27—1.71). Estimates reveal that across all samples, women are far less likely to bribe (β = −0.191, *p* = 0.007). In accordance with our expectations, WtB strictly and transitively decreases with darkening shades of bribery (white against black: Δβ = |0.265|; *p* < 0.000).

Robust linear regression estimation with both WtB as the combined measure as well as its four components separately (*likelihood*, *justification*, *affect*, and *mistake*) as the dependent variable and including overall PSM’s and SVO’s direct effect (see Models II in Table 2) shows, first, that the relationship of PSM and WtB is negative throughout (e.g., β = −0.013 for composite WtB) but not statistically significantly so (e.g., *p* = 0.056 for composite WtB) with the exception of *mistake*. Regarding the latter, higher PSM is significantly and positively linked with respondents’ awareness that engaging in bribes would be a mistake (β = 0.103, *p* = 0.019). Sign-wise, this series of findings is fully in line with H1. Second, we observe that the relationship between SVO and WtB is consistently positive across all bribery dimensions (β = |0.167—0.196|), except for the negative estimate for *mistake* (β = 0.172), and statistically significant (*p* = 0.001—0.026), which consistently goes against with H2. Third, the multivariate regression analyses including interaction effects (see Models III in Table 2) reveal only mild and statistically insignificant interaction effects between PSM and SVO, both on WtB and, fully consistently so, across all four WtB items (β = |0.026—0.038|; *p* = 0.000—0.006). This implies absence of any support for H3.

Finally, regarding H4, we note that the correlation between PSM and SVO is substantial and statistically significant, but negative (*r* = −0.412; *p* < 0.000). We estimate partial mediation by conducting a full-blown moderated mediation analysis. We do so by running a structural equation model (SEM), including all control variables, using maximum likelihood parameter estimation, clustering the data at the individual level of respondents for conditional contributions to the observations. The estimated model has a good model-to-data fit (CFI = 0.165; TLI = −1.027; RMSEA = 0.335; Chi^2^ (7) = 39,766.61). The main findings are summarized in Figure 2.^1^

Regarding *direct effects*, high scores on SVO (SVO high) are related strongly and negatively to PSM (β = −0.705, *p* < 0.000), PSM is negatively but insignificantly associated with greater willingness to engage in bribery (β = −0.002, *p* > 0.05), and SVO has a weak and positive but statistically significant direct effect on WtB (β = 0.028, *p* = 0.001).^2^ As far as *indirect* effects are concerned, we hypothesized that the relationship between high SVO and low WtB is mediated in the form of an indirect and negative effect on WtB through PSM. This partial mediation is not supported, though, because the standardized indirect coefficient is not statistically significant (β = 0.002, *p* > 0.05). Moreover, the PSM^∗^SVO interaction is positive but insignificant (β = 0.000, *p* > 0.10). Consequently, we cannot provide any support for H4. Overall, our SEM analyses are sign-consistent but insignificant regarding H1, provide evidence against H2, and are unsupportive as to H3 and H4. So, as far as H1, H2, and H3 is concerned, the SEM findings are fully in line with the results of the robust linear regression.

## Discussion

We first interpret the pattern of findings in line with the guidelines of Meyer et al. (2017). In their editorial essay in the *Journal of International Business Studies*, they argue that a blind focus on *p*-value thresholds is misguided: Not only is any *p*-value threshold arbitrary, but also are effect sizes equally, if not more, important. First, the findings confirm that PSM is directly and negatively associated with peoples’ willingness to engage in bribery, but only significantly so regarding respondents’ awareness that engaging in a bribe would be a *mistake*. For all other WtB items, the relationship is not statistically significant, and small but positive (and hence sign-supportive). Note that we find that the different underlying PSM dimensions have hardly any significant effect on WtB, with the only exception being CPI (β = −0.039, *p* = 0.087; see Supplementary Appendix A.4.1 for additional analyses) mediated through *justification*. Second, in contrast, SVO’s relationships with WtB and the four underlying items are sign-consistent and statistically significant, which means that the people with higher SVO are more willing to engage in bribery. This set of relationships is consistent across all three country samples. Third, furthermore, we find a negative correlation between the two forms of pro-sociality (PSM and SVO), and no evidence for mediation or moderation. All in all, we have sign-consistent support for H1, evidence that goes against H2, and no support for H3 and H4.

Looking at this pattern of findings, two specifically represent puzzles that require further interpretation, as we expected the opposite: Why is SVO negatively related with PSM, and positively with WtB? Given the fact that the association of the *trait*-based *other*-regarding motive SVO with WtB is positive and that of the *attitude-*based *society*-regarding motive of PSM with WtB is negative, we may suspect that this has to do with the different nature of the underlying motivational system. Indeed, because of their very different natures and roots, we know from prior work in motivational theory that differently-rooted motives often are incongruent (see, e.g., Hermans et al. (2017), focusing on explicit versus implicit motives). We speculate that the external social norm-like antecedents of an individual’s PSM may be suppressed by the internal intrinsic motive-like drivers of SVO. Specifically, this might imply that SVO’s intrinsic motivation to help specific and concrete others may overrule PSM’s norm-driven “prescription” to not do so for the sake of serving a more abstract public interest. Of course, we cannot be sure that our interpretation is correct. And of course, the negative finding for SVO might be a sample-specific statistical artifact that will disappear in another sample. However, given that we replicate the negative SVO-WtB and SVO-PSM associations in all three country samples, we suspect that we may need an alternative theory, such as the one suggested here, which has to be examined in future work.

The three replicative country studies were conducted in three West-European countries in which the tolerance for bribery is perceived as relatively low (Wilhelm, 2002; Transparency International, 2021), implying that the institutional environment is unlikely to be a distinctive factor. Indeed, the analysis does reveal only marginally small differences between samples. In comparison to the Belgian sample that served us as the arbitrary reference category, faced with the same bribery scenario, respondents from Germany found it easier to derive contextual justification that leads to a higher willingness to engage in bribery. In contrast, Dutch respondents were much less likely to justify bribing, *ceteris paribus*. This relates to research by Montinola and Jackman (2002), Davis and Ruhe (2003), and Achim (2016), pointing out that the factors that stimulate bribery can well be located at the greater macro-level. Cultural and institutional environments frame individual behavior by shaping expectations, attitudes, and norms that are learned through socialization processes (Morgan, 2006), and which are very specific to country cultures. Taking Hofstede’s (1984) classical set of cultural dimensions, Germany scores relatively low on *indulgence* compared with Belgium and the Netherlands, which are very similar in this respect (Hofstede and McCrae, 2004). Individuals socialized in cultures with low indulgence are normally restrained by strict social norms, and have a stronger tendency of responding to incentives to break these rules, which might manifest in monetary but also emotional bribes (Achim, 2016). This may explain why German respondents highly driven by compassion are triggered to act more pro-social when being confronted with pro-self behavior as a way of engaging with the person in need by showing generosity. In our context, this may translate into a higher willingness to engage in bribery among our German sample.

Furthermore, respondents are more likely to accept the use of bribery if they perceive the vignette treatment as more realistic. This is a striking finding, contradicting extant theory in at least two ways. First, despite its great potential, experimental vignettes research has often been criticized for its limited external validity on the argument that vignettes studies are too abstract. Hence, such studies might not capture actual behavioral intent under real-life conditions (Bouwman and Grimmelikhuijsen, 2016). Second, respondents would be expected to be more willing to demonstrate socially acceptable behavior in a realistic setting, implying a lower likelihood of accepting bribery since bribery is subject to stigmatization, certainly in our set of three Western-European countries (Tourangeau and Yan, 2007). Empirical evidence regarding honesty in questionnaire responses by Hughes and Huby (2004) and Kreuter et al. (2008) imply that realism in vignette treatments can have a moderating effect on the social desirability response bias, but only if the treatment scenarios are directly related to respondents’ own experiences. Consequently, the realism finding can be interpreted as evidence that well-conducted and thoroughly pre-tested vignettes can be of substantial value for behavioral experimental research that tries to tackle the essential and delicate issues.

As said, PSM is a manifestation of the society-regarding attitude-based motivational system, and SVO the manifestation of the other-regarding trait-based motivational system. Here, in the context of bribery research, we enter unknown territory. We are not aware of any work examining the effect of such differently-rooted motives, in isolation and in tandem, on bribery. However, like any study, ours is subject to limitations. First, we only focused on bribery in a specific student-teacher setting, while the primary motives of public officials and business employees to engage in bribery acts might be rooted in different convictions, limiting the generalizability of the findings. Yet, prior studies such as Gorsira et al. (2018) have shown that primary motives from public officials and business employees to engage in acts of bribery were virtually identical. Second, measuring respondents’ context-dependent intention to bribe, this study does not examine real-world behavior, but reveals behavioral intent that might prime real-world behavior. Although the realism of vignettes was pretested and controlled for, further work is needed in the context of real-world behavior. Specifically, given the social desirability bias, the acceptability of bribery in this study might be underreported. Third, the study was conducted, by design, in three countries with similar levels of bribery. Nevertheless, our data reveal differences between countries that point toward a more complex interaction between the macro-institutional context and the micro-constructs of PSM (dimensions) and SVO. Given that even in a multi-site study in three countries selected for *not* being very different, interesting differences exist, we strongly call for the replication of this study in countries that *are* very different. Doing so will shed light on the impact of macro institutional variations on the likelihood of bribery, as well as on the interaction of macro, meso, and micro determinants of such behavior.

## Conclusion

The motivation of this study was to explore the connection between bribery, PSM, and SVO. Using a multi-site replication approach, the results of this study not only show that PSM is significantly related to bribery, but also that SVO is an antecedent of society-regarding PSM and that individuals with a higher orientation toward social pro-other values are more likely to accept the use of bribery. The results of this study contribute to the broad discourse on both PSM and corruption by illustrating how relevant concepts such as PSM and SVO in isolation and in tandem affect bribery willingness. Furthermore, our study comes with a few methodological advancements. First, the research design proved to be robust by replicating the study in three different countries. Second, the quasi-experimental design provides the opportunity to find out to what extent bribery is contextually dependent. Third, the vignettes and four-item willingness-to-bribe measure that were developed within the scope of this research are now validated in three different country settings and can, thus, be used in future research.

Furthermore, the findings of this study are particularly relevant for practice. Practitioners seeking to diminish the likelihood of bribery should prefer to employ people with low social value orientation, who are not so much driven by pro-other motives, and who hold a high commitment to the public interest. However, human resource managers in the private and public sector should also recognize that—depending on the greater cultural context, and perhaps counterintuitively—high compassion with others and a tendency to put others’ goals first can also lead to more susceptibility to bribery. Furthermore, practitioners should keep an open eye on more subtle forms of bribery such as emotional pleading or offering a helping hand, because people are much more susceptible to these ‘white’ and “gray” forms of bribery than to the classic brown envelop.

We can identify a few avenues for future research. First, the study calls for further replication in order to find out to what extent the greater institutional context primes individual behavior under varying levels of PSM and SVO, in isolation and in tandem. Second, this study only focused on a pro-self form of bribery. However, in line with a recent stream of conceptual research (e.g., Schott and Ritz, 2018), the findings illustrate that there might be a connection with pro-social forms of bribery as well. Third, further studies are needed to understand whether the effects of PSM and SVO revealed in the current study differ if the treatment scenario was about being offered a bribe and deciding whether or not to accept this bribe (in contrast to offering the bribe oneself). Therefore, future studies are encouraged to investigate whether PSM and SVO, in isolation and in tandem, might also have an effect on pro-social behavior such as pro-social rule-breaking.

A final future research issue relates to the fundamental question as to the very nature of PSM. In the current study, we used an explicit survey measure of PSM. Theory distinguishes between two types of motivational systems: an implicit system operating unconsciously, and an explicit system that functions consciously. Implicit motives develop during early childhood on the basis of non-verbal, affective experiences, and explicit motives are acquired after the development of language under the influence of explicit instructions of the social and cultural environment (McClelland and Pilon, 1983; Kasser et al., 2002). Moreover, importantly, implicit and explicit motives fundamentally differ in their behavioral impact. Implicit motives are associated with spontaneous, uncontrolled behavior, and effort-related task performance; explicit human motives drive behavior that is subject to conscious thought and deliberation, such as self-reflective appraisals and deliberate choices (Perugini et al., 2010; Schultheiss and Brunstein, 2010). Survey measures of motives are explicit, as people consciously respond to items. Implicit motives must be captured by non-explicit measures (e.g., Bing et al., 2007; Payne and Gawronski, 2010; Lang et al., 2012; Runge and Lang, 2019; Runge et al., 2020). An example of such an implicit measure is the (Brief) Implicit Association Test, or (B)IAT, which Slabbinck and van Witteloostuijn (2020) used to develop an implicit PSM measure, revealing that the explicit survey measure of PSM is indeed very different from their implicit BIAT counterpart. In future research, we hope to explore how explicit and implicit motivational measures might impact the likelihood of bribery differently.

## Data Availability Statement

The raw data supporting the conclusions of this article will be made available by the authors, without undue reservation upon request.

## Ethics Statement

Ethical review and approval was not required for the study on human participants in accordance with the local legislation and institutional requirements. The patients/participants provided their written informed consent to participate in this study.

## Author Contributions

All authors listed have made a substantial, direct and intellectual contribution to the work, and approved it for publication.

## Conflict of Interest

The authors declare that the research was conducted in the absence of any commercial or financial relationships that could be construed as a potential conflict of interest.

## Footnotes

1 Detailed SEM results, including those per WtB item, are available upon request.

2 Detailed results available upon request.
