# Erratum: Bribery and the Role of Public Service Motivation and Social Value Orientation: A Multi-Site Experimental Study in Belgium, Germany and the Netherlands

**DOI:** 10.3389/fpsyg.2021.730179

**Published:** 2021-07-14

**Authors:** Frontiers Production Office

**Affiliations:** Frontiers Media SA, Lausanne, Switzerland

**Keywords:** bribery, corruption, social value orientation (SVO), public service motivation (PSM), multi-site design

Due to a production error, the title is missing the following part: “of Public Service Motivation” The title should read: “Bribery and the Role of Public Service Motivation and Social Value Orientation: A Multi-Site Experimental Study in Belgium, Germany and the Netherlands”. The publisher apologizes for this mistake.

The original article has been updated.

