# Supplementary material for: Bribery and the Role of Public Service Motivation and Social Value Orientation: A Multi-Site Experimental Study in Belgium, Germany and the Netherlands
Source: Front Psychol. 2021 Jun 7;12:655964. doi: 10.3389/fpsyg.2021.655964 (PMC8215125; doi:10.3389/fpsyg.2021.655964)
Supplement: Supplementary file 1 [file Data_Sheet_1.DOCX]

# **APPENDIX** (Supplementary Online Material)

## Appendix A.1: Vignette stimuli

*Extensive codebook in Dutch and German available upon request.*

**General introduction to bribery scenarios [all study participants]:**

| Please imagine that you are a first-year student again who has just received his results for the end of term exams. You passed all courses but one. You failed to pass one very difficult course you really do not want to redo. The consequence is that your prerequisites in the next academic year become compromised and you are unable to participate in other courses so that chances are real that you will not succeed to obtain your degree within the foreseen four years.  Meanwhile, you informed the assistant of this course in order to receive written feedback. This feedback indicates that you achieved 9.4/2. You know that if you would have scored 9.5/20, your result would be rounded off to 10/20 so that you would have passed the exam and the study program of the first year would have been accomplished.  What would you do in the following two situations? |
| --- |

**Vignettes:** Study participants randomly received two out of three vignette treatments (A, B, C):

| **A** | **“Emotional plea”: white bribery**  ‘You make an appointment with the lecturer of this course and inform him about your situation. Rumour goes that, in the past, the lecturer let himself be influenced in a personal conversation. After you became emotional, you ask the lecturer if he, due to the circumstances, would consider being a little bit milder with regard to your result so that you can finally succeed in this course. Your future relies on this.’ |
| --- | --- |
| **B** | **“Car mechanic”: grey bribery**  ‘You make an appointment with the lecturer of this course and inform him about your situation. Rumour goes that, in the past, the lecturer let himself be influenced in a personal conversation. The lecturer is a little bit too late and apologizes. He experienced car trouble, which is very unfortunate for the reason that he has to leave for an important conference tomorrow. However, your father is a car mechanic. You offer your lecturer to repair the car, free of charge and with the highest priority, on the condition that your result is reconsidered.’ |
| **C** | **“Brown envelop”: black bribery**  ‘You make an appointment with the lecturer of this course and inform him about your situation. Rumour goes that, in the past, the lecturer let himself be influenced in a personal conversation. You ask him to reconsider your score and therefore offer him an envelope with €500 in exchange.’ |

**Willingness to bribe:** Each vignette treatment was followed by these five Likert-type questions:

| “The following statements relate to the preceding scenario. Please indicate to what extent you agree with the following statements:   1. This scenario appears realistic. [1=totally disagree; 4=totally agree] 2. How likely do you think you will try to influence your lecturer in an attempt to have your grade adjusted upward? [1=very unlikely; 5=very likely] 3. How justified do you find influencing your lecturer in an attempt to have your grade adjusted upward? [1=very unjustified; 5=very justified] 4. How would you feel about influencing your lecturer in an attempt to have your grade adjusted upward? [1=very uncomfortable; 5=very comfortable] 5. I think that influencing my lecturer in an attempt to have my grade adjusted upward in this situation would be a mistake. [1=totally disagree; 5=totally agree].” |
| --- |

## Appendix A.2: PSM scale

**Dutch**

| APS 1: | Ik ben geïnteresseerd in overheidsbeleid dat goed is voor mijn land of de gemeenschap waartoe ik behoor. |
| --- | --- |
| APS 2: | Ik vind het leuk om mijn mening te delen over het beleid van de overheid. |
| APS 3: | Zien dat mensen voordeel hebben van het overheidsbeleid waar ik intens bij betrokken ben geweest, zou me veel voldoening geven. |
| CPV 1: | Ten dienste staan van het algemeen belang beschouw ik als mijn burgerplicht. |
| CPV 2: | Betekenisvol werk in het algemeen belang is erg belangrijk voor mij. |
| CPV 3: | Ik zou als ambtenaar het liefst doen wat best is voor het algemeen belang, zelfs als dat mijn persoonlijke belangen schaadt. |
| COM 1: | Het is moeilijk voor mij om mijn gevoelens te beheersen als ik mensen in armoede zie. |
| COM 2: | Dagelijkse gebeurtenissen herinneren mij er vaak aan hoe afhankelijk we zijn van elkaar. |
| COM 3: | Ik voel mee met de benarde situatie van de kansarmen. |
| SS 1: | Een verschil maken in de maatschappij betekent meer voor mij dan persoonlijke prestaties. |
| SS 2: | Ik ben bereid om grote offers te brengen voor het welzijn van de maatschappij. |
| SS 3: | Ik geloof dat plicht belangrijker is dan eigenbelang. |

Measures of fit: Chi² (54)=21,309; *p*<.000; GFI=.691; RMSEA=.156; CFI=.692.

**English** (original scale by Kim et al. 2013)

| APS 1: | I am interested in those public programs that are beneficial for my country or the community I belong to. |
| --- | --- |
| APS 2: | Sharing my views on public policies with others is attractive to me. |
| APS 3: | Seeing people getting benefits from a public program where I would have been deeply involved in would bring me a great deal of satisfaction. |
| CPV 1: | I consider public service my civic duty. |
| CPV 2: | Meaningful public service is very important to me. |
| CPV 3: | I would prefer seeing public officials do what is best for the whole community even if it harmed my interests. |
| COM 1: | It is difficult for me to contain my feelings when I see people in distress. |
| COM 2: | I am often reminded by daily events how dependent we are on one another. |
| COM 3: | I feel sympathetic for the plight of the unprivileged. |
| SS 1: | Making a difference in society means more to me than personal achievements. |
| SS 2: | I am prepared to make enormous sacrifices for the good of the society. |
| SS 3: | I believe in putting duty before self. |

Measures of fit (Kim et al. 2012): Chi² (98)=1,266.6; *p*<.05; RMSEA=.041; CFI=.989.

**German**

| APS 1: | Ich interessiere mich für öffentliche Programme, die dem Land oder der Gemeinde, in dem bzw. in der ich lebe, zu Gute kommen. |
| --- | --- |
| APS 2: | Ich tausche mich gerne mit anderen über meine politischen Vorstellungen aus. |
| APS 3: | Es würde mir sehr gefallen, wenn es Menschen durch mein Engagement für das Gemeinwohl besser ginge. |
| CPV 1: | Ich sehe die Arbeit im öffentlichen Dienst als meine Bürgerpflicht. |
| CPV 2: | Eine sinnvolle Arbeit im öffentlichen Sektor ist wichtig für mich. |
| CPV 3: | Ich bevorzuge es, wenn Amtsträger ihr Handeln am Wohle der Gesellschaft ausrichten, selbst wenn es meinen eigenen Interessen zuwiderläuft. |
| COM 1: | Es ist schwer für mich, meine Gefühle zu unterdrücken, wenn ich Menschen in Not sehe. |
| COM 2: | Alltägliche Dinge erinnern mich oft daran, wie sehr wir alle aufeinander angewiesen sind. |
| COM 3: | Ich habe Mitgefühl mit den Benachteiligten der Gesellschaft. |
| SS 1: | Etwas für die Gesellschaft zu erreichen, ist mir wichtiger als persönliche Erfolge. |
| SS 2: | Ich bin bereit, große Opfer für das Gemeinwohl zu bringen. |
| SS 3: | Ich bin überzeugt, dass die eigenen Interessen den Aufgaben und Pflichten untergeordnet werden sollten. |

Measures of fit: Chi² (54)=17,291; *p*<.000; GFI=.777; RMSEA=.143; CFI=.778.

**REFERENCE**

Kim, S., Vandenabeele, W., Wright, B. E., Andersen, L. B., Cerase, F. P., Christensen, R. K., Desmarais, C., Koumenta, M., Leisink, P., Liu, B., Palidauskaite, J., Pedersen, L. H., Perry, J. L., Ritz, A., Taylor, J., De Vivo, P. (2012). Investigating the Structure and Meaning of Public Service Motivation across Populations: Developing an International Instrument and Addressing Issues of Measurement Invariance, Journal of Public Administration Research and Theory 23(1), 79-102.

## Appendix A.3: Task items of Social Value Orientation (SVO)

| Trial No. | Item No. | Own pay-out | Other person’s pay-out | Δ^a^ | Choice motive |
| --- | --- | --- | --- | --- | --- |
| 1 | 1 | 480 | 80 | 400 | competitive |
|  | 2 | 540 | 280 | 260 | individualistic |
|  | 3 | 480 | 480 | 0 | prosocial |
| 2 | 4 | 560 | 300 | 260 | individualistic |
|  | 5 | 500 | 500 | 0 | prosocial |
|  | 6 | 500 | 100 | 400 | competitive |
| 3 | 7 | 520 | 120 | 400 | competitive |
|  | 8 | 520 | 520 | 0 | prosocial |
|  | 9 | 580 | 320 | 260 | individualistic |
| 4 | 10 | 500 | 100 | 400 | competitive |
|  | 11 | 560 | 300 | 260 | individualistic |
|  | 12 | 490 | 490 | 0 | prosocial |
| 5 | 13 | 560 | 300 | 260 | individualistic |
|  | 14 | 500 | 500 | 0 | prosocial |
|  | 15 | 490 | 90 | 400 | competitive |
| 6 | 16 | 500 | 500 | 0 | prosocial |
|  | 17 | 500 | 100 | 400 | competitive |
|  | 18 | 570 | 300 | 270 | individualistic |
| 7 | 19 | 510 | 510 | 0 | prosocial |
|  | 20 | 560 | 300 | 260 | individualistic |
|  | 21 | 510 | 110 | 400 | competitive |
| 8 | 22 | 550 | 300 | 250 | individualistic |
|  | 23 | 500 | 100 | 400 | competitive |
|  | 24 | 500 | 500 | 0 | prosocial |
| 9 | 25 | 480 | 100 | 380 | competitive |
|  | 26 | 490 | 490 | 0 | prosocial |
|  | 27 | 540 | 300 | 240 | individualistic |

*Notes*: Hypothetical pay-out amounts in €; Implicit measure developed by Bogaert et al. (2012); and ^a^ Δ = pay-out difference.

## Appendix A.4: Details of dependent variable validation

Table A.4.1 reports the results of the exploratory factor analysis and unique variances for each item as well as the respective Kaiser-Meyer-Olkin (*KMO*) measure of sample adequacy. *KMO* mean values range between .82 (Germany) to .83 (Belgium and the Netherlands) which indicates meritoriously high sample adequacy (Kaiser, 1974).

Table A.4.1: Results of factor analysis of dependent variable by country sample

|  | **Belgium** | | | **Germany** | | | **The Netherlands** | | |
| --- | --- | --- | --- | --- | --- | --- | --- | --- | --- |
| **Factor item** | Factor 1 | U | KMO | Factor 1 | U | KMO | Factor 1 | U | KMO |
| Likelihood | .80 | .37 | .84 | .75 | .43 | .85 | .83 | .30 | .83 |
| Justification | .86 | .26 | .79 | .86 | .26 | .77 | .88 | .23 | .78 |
| Affect | .78 | .39 | .85 | .79 | .38 | .83 | .81 | .34 | .85 |
| Mistake ^a^ | .76 | .43 | .87 | .73 | .47 | .84 | .71 | .50 | .90 |
| Eigenvalue | 2.56 |  |  | 2.46 |  |  | 2.63 |  |  |
| Bartlett Chi² (6) | 946.35 |  |  | 866.76 |  |  | 914.01 |  |  |
| *p* | .000 |  |  | .000 |  |  | .000 |  |  |
| Cronbach’s α | .880 |  |  | .865 |  |  | .885 |  |  |

*Notes*: *U* = uniqueness; *KMO* = Kaiser-Meyer-Olkin measure; and ^a^ reversed item.

Prior to factor analysis, Bartlett’s test for sphericity was conducted to test whether factor items are inter-correlated which is a prerequisite for factor analysis. As expected from the analysis of the correlation matrix of the items (see table A.4.2), the derived factor model scored very high across all country samples and the significant *Chi*²-testing results of Bartlett’s test (*Chi²*(6): 866.76–945.35, *p<*.000) indicate that factor items are interrelated and should load onto the same factor(s). The factor analysis results show that the four items strongly and significantly load onto one single factor. This finding is, again, stable across all three country samples, indicating high internal and external validity of the developed construct of ‘willingness to bribe’ (WtB) with its four components.

Table A.4.2: Correlations between factor items of dependent variable by study

|  | **Study 1 (BEL)** | | | |  | **Study 2 (GER)** | | | |  | **Study 3 (NL)** | | | |
| --- | --- | --- | --- | --- | --- | --- | --- | --- | --- | --- | --- | --- | --- | --- |
| **Factor item** | a | b | c | d |  | a | b | c | d |  | a | b | c | d |
| a Likelihood | 1 |  |  |  |  | 1 |  |  |  |  | 1 |  |  |  |
| b Justification | .72 | 1 |  |  |  | .68 | 1 |  |  |  | .77 | 1 |  |  |
| c Affect | .64 | .71 | 1 |  |  | .64 | .71 | 1 |  |  | .69 | .74 | 1 |  |
| d Mistake ^a^ | .62 | .68 | .61 | 1 |  | .54 | .69 | .56 | 1 |  | .60 | .64 | .59 | 1 |

*Notes*: All correlations significant with *p<*.000; and ^a^ reversed item.

Item uniqueness (*U*) is usually regarded as a measure of the percentage of variance for the item that is not explained by the common factors. Values of *U*=.6 are considered as high. In our analysis, uniqueness values range from *U*=.23 to .5. Since items with lower uniqueness matter less for explaining the variance observed, this means that, firstly, *justification* was relatively less important in explaining the variance observed than items with relatively higher uniqueness values, e.g. *mistake (reverse)* ranging from *U*=.43 to .50 or affect (*U*=.34 – .38). Secondly, across all three samples, items are in a relatively stable and narrow range which indicates only subtle differences between samples and further substantiates the measure’s internal validity in measuring one underlying construct and its robustness against country-specific influences, indicating high external validity. Because of the high inter-correlation and the strong factor model fit, no item was excluded and the final dependent variable of this study is created by arithmetically sum-scoring the four indicators *likelihood*, *justification*, *affect*, and *mistake* (reversed).

**REFERENCE**

Kaiser, Henry F. (1974). An index of factor simplicity. *Psychometrika* *39*(1), 31-36.

## Appendix A.5: Additional analysis with split PSM dimensions

**Table A.5.1**: Main effects estimates on WtB dimensions by PSM dimensions (pooled data)

|  | *Likelihood* | *Justification* | *Affect* | *Mistake* | *WtB* |
| --- | --- | --- | --- | --- | --- |
| PSM 1: APM | -.006 | -.012 | -.009 | .034 | -.006 |
|  | (.029) | (.029) | (.028) | (.037) | (.017) |
| PSM 2: CPI | -.058 | -.082* | -.051 | .042 | -.039† |
|  | (.039) | (.040) | (.035) | (.047) | (.023) |
| PSM 3: COM | .024 | .026 | -.008 | -.016 | .004 |
|  | (.033) | (.035) | (.033) | (.038) | (.019) |
| PSM 4: SS | .001 | .026 | .007 | .034 | .022 |
|  | (.032) | (.033) | (.030) | (.040) | (.019) |
| SVO | .198** | .184** | .169** | -.180* | .136*** |
|  | (.067) | (.066) | (.062) | (.077) | (.040) |
| Age | .005 | .018* | .004 | -.029** | .000 |
|  | (.007) | (.008) | (.007) | (.010) | (.004) |
| Female | -.127† | -.169** | -.239*** | .118 | -.129*** |
|  | (.065) | (.062) | (.058) | (.073) | (.037) |
| White bribery | .436*** | .388*** | .161* | -.518*** | .152*** |
|  | (.077) | (.074) | (.071) | (.091) | (.044) |
| Grey bribery | *- reference category -* | | | | |
|  |  |  |  |  |  |
| Black bribery | -.120 | -.015 | -.021 | -.081 | -.098* |
|  | (.080) | (.072) | (.064) | (.086) | (.0422) |
| Germany | -.107 | .151† | -.088 | -.101 | -.030 |
|  | (.081) | (.082) | (.071) | (.101) | (.046) |
| Belgium | *- reference category -* | | | | |
|  |  |  |  |  |  |
| The Netherlands | .019 | -.166* | -.034 | .089 | -.032 |
|  | (.075) | (.067) | (.069) | (.079) | (.043) |
| Intercept | 1.824*** | 1.525*** | 1.981*** | 4.371*** | 2.137*** |
|  | (.300) | (.308) | (.319) | (.387) | (.176) |
| *N* | 622 | 622 | 622 | 622 | 622 |
| *F* | 8.53 | 9.44 | 5.38 | 7.21 | 7.27 |
| *p* | .000 | .000 | .000 | .000 | .000 |
| *VIF ^a^* | 1.45 | 1.45 | 1.45 | 1.45 | 1.45 |
| *R^2^* | .056 | .067 | .045 | .062 | .063 |
| *RMSE* | 1.131 | 1.060 | .892 | 1.216 | .570 |

*Notes*: Linear regression modelling clustered by respondent for conditional contribution, robust standard errors in parentheses; ^a^ Mean variance inflation factor (*VIF*), all *VIF*≤1.95; and † *p*<.10, * *p*<.05, ** *p*<.01, and *** *p*<.001.

## Appendix A.6: Additional Main Effect Analyses

**Table A.6.1**: Main effects estimates (country data)

|  | *Likelihood* | | |  | *Justification* | | |  | *Affect* | | |  | | *Mistake* | | | |  | *WtB* | | |
| --- | --- | --- | --- | --- | --- | --- | --- | --- | --- | --- | --- | --- | --- | --- | --- | --- | --- | --- | --- | --- | --- |
|  | BEL | GER | NL |  | BEL | GER | NL |  | BEL | GER | NL |  | BEL | | GER | | NL |  | BEL | GER | NL |
| PSM | -.094 | -.035 | .010 |  | .006 | -.103 | -.028 |  | -.086 | -.076 | -.039 |  | .116 | | .125 | | .085 |  | -.026 | -.023 | .006 |
|  | (.071) | (.054) | (.066) |  | (.058) | (.064) | (.063) |  | (.093) | (.059) | (.063) |  | (.071) | | (.082) | | (.072) |  | (.038) | (.031) | (.038) |
| SVO | .025 | .269* | .258† |  | .168 | .143 | .115 |  | .192† | .118 | .134 |  | -.137 | | -.107 | | -.113 |  | .099 | .148* | .122 |
|  | (.117) | (.120) | (.132) |  | (.113) | (.129) | (.116) |  | (.114) | (.102) | (.122) |  | (.121) | | (.151) | | (.135) |  | (.074) | (.066) | (.078) |
| Age | -.002 | .019† | -.022* |  | .002 | .032** | -.011 |  | .003 | .016† | -.013 |  | -.012 | | -.035* | | -.021 |  | -.002 | .007 | -.016* |
|  | (.019) | (.011) | (.010) |  | (.018) | (.011) | (.010) |  | (.020) | (.009) | (.011) |  | (.019) | | (.014) | | (.023) |  | (.012) | (.005) | (.007) |
| Female | -.115 | -.078 | -.108 |  | -.194* | .023 | -.210* |  | -.300** | -.070 | -.299** |  | .111 | | -.088 | | .217† |  | -.161** | -.064 | -.136* |
|  | (.107) | (.121) | (.116) |  | (.092) | (.130) | (.101) |  | (.096) | (.102) | (.104) |  | (.106) | | (.157) | | (.124) |  | (.062) | (.066) | (.067) |
| White bribery | .651*** | .356* | .241† |  | .476*** | .411** | .322** |  | .201 | .102 | .168 |  | -.727*** | | -.317 | | -.481** |  | .214** | .140† | .120 |
|  | (.124) | (.144) | (.132) |  | (.118) | (.150) | (.113) |  | (.123) | (.122) | (.126) |  | (.126) | | (.192) | | (.157) |  | (.078) | (.077) | (.080) |
| Grey bribery |  |  |  |  |  |  |  |  | *- reference category -* | | |  | |  | |  |  |  |  |  |  |
|  |  |  |  |  |  |  |  |  |  |  |  |  |  | |  | |  |  |  |  |  |
| Black bribery | -.048 | .042 | -.363** |  | -.073 | .076 | -.061 |  | -.052 | .105 | -.082 |  | -.153 | | -.190 | | -.007 |  | -.116 | -.016 | -.158* |
|  | (.140) | (.136) | (.139) |  | (.111) | (.149) | (.117) |  | (.114) | (.112) | (.111) |  | (.134) | | (.167) | | (.141) |  | (.073) | (.074) | (.074) |
| Intercept | 2.104*** | 1.218** | 2.452*** |  | 1.498** | 1.448** | 2.054*** |  | 2.105*** | 1.533** | 2.279*** |  | 4.127*** | | 4.258*** | | 4.175*** |  | 2.195*** | 1.840*** | 2.419*** |
|  | (.554) | (.450) | (.484) |  | (.454) | (.552) | (.425) |  | (.600) | (.473) | (.467) |  | (.566) | | (.634) | | (.678) |  | (.301) | (.279) | (.293) |
| *F* | 8.58 | 2.60 | 6.78 |  | 7.81 | 3.63 | 4.00 |  | 5.34 | 1.43 | 4.55 |  | 8.13 | | 1.82 | | 3.45 |  | 7.12 | 2.26 | 5.50 |
| *p* | .000 | .019 | .000 |  | .000 | .002 | .000 |  | .000 | .206 | .000 |  | .000 | | .097 | | .003 |  | .035 | .040 | .000 |
| *N* | 193 | 192 | 198 |  | 193 | 192 | 198 |  | 193 | 192 | 198 |  | 193 | | 192 | | 198 |  | 193 | 192 | 198 |
| *VIF* | 1.20 | 1.21 | 1.27 |  | 1.20 | 1.21 | 1.27 |  | 1.20 | 1.21 | 1.27 |  | 1.20 | | 1.21 | | 1.27 |  | 1.20 | 1.21 | 1.27 |
| *R^2^* | .085 | .037 | .058 |  | .08 | .037 | .051 |  | .073 | .025 | .053 |  | .088 | | .028 | | .051 |  | .092 | .038 | .072 |
| *RMSE* | 1.100 | 1.088 | 1.177 |  | .959 | 1.209 | .938 |  | .869 | .857 | .920 |  | 1.121 | | 1.310 | | 1.188 |  | .559 | .555 | .579 |

*Notes*: Linear regression modelling clustered for conditional contribution, robust standard errors in parentheses; † *p*<.10, * *p*<.05, ** *p*<.01, and *** *p*<.001.
